# Participants’ Experiences of Sex Offender Treatment: Searching for Desisting Narrative Identities

**DOI:** 10.1177/0306624X241228231

**Published:** 2024-02-05

**Authors:** Stina Lindegren

**Affiliations:** 1 Department of Social Work, Uppsala University, Uppsala, Sweden

**Keywords:** Narrative identity, Turning points, Desistance, Sexual offender treatment, Early desistance, Active responsibility

## Abstract

Life-course criminology has demonstrated the importance of social relationships and life transitions to understand desistance. Yet, individuals convicted of sexual offenses seem to differ in terms of turning points, where treatment is suggested as salient to their desistance processes. Drawing on 13 teller-focused interviews with adult male incarcerated participants in a new Swedish sex offender program, this paper examines the treatment experience and the under-explored aspect of early desistance, as well as the role of society and social relations in the treatment process, through a practice-oriented lens. The thematic analysis suggests participants started developing desisting narrative identities where micro turning points in treatment contributed to offenses being explained and re-integrated into a sense of the self as an acceptable person. The process, which also enabled active responsibility, seemed to be facilitated by a holistic, person-centered treatment environment. Nonetheless, continued desistance requires adequate attention to social support and stigma management post-release.

Research on desistance is central in life course criminology and a growing interest within the study of individuals convicted of sexual offenses (ISO) (McCartan & Richards, 2021). Desistance can occur naturally during the life-course (Moffitt, 1993) or as a result of criminal justice interventions or treatment programs (Harris, 2021). Distinctions are made between *primary* and *secondary desistance*, i.e. the cessation of criminal acts, respectively, long-term prosocial identity transformations (Maruna & Farrall, 2004; McNeill et al., 2010). *Early desistance* is used to describe the transition from primary to secondary desistance (King, 2013).

Desistance processes of ISOs share similarities but also appear to differ in some ways in comparison to non-sexual offenders (Farmer et al., 2015; Harris, 2021; McAlinden et al., 2017). The significance of employment and relationships does not seem to entail a linear association to a non-offending identity among ISOs. Instead, the importance of treatment seems more pronounced in desisting ISOs’ accounts in comparison to non-sexual offenders, which calls for more research into the role of treatment in ISOs’ desistance processes (Farmer et al., 2015). Desistance research acknowledges the significance of treatment for ISOs but has rarely engaged in a deeper examination of how treatment and practitioners might support early desistance (cf. King, 2013) or the impact of the ISOs’ family and friends on treatment experience and vice versa, as well as societal influence.

Sex offender treatment research and policy have investigated and debated the issue of risk factors and effectiveness on recidivism for decades, but they have not sufficiently engaged in the question of *how* ISOs manage to change their lives, i.e., desistance processes (Harris, 2021; McCartan & Richards, 2021). This is achieved, for instance, by qualitatively studying treatment participant experiences. The study of desistance in the ISO population is rare (Farmer et al., 2015; Harris, 2021) and studies focusing specifically on ISOs’ treatment experiences rarely engage much with process-oriented theoretical desistance frameworks. Rather, many of these studies are consumer-oriented (how participants experience certain components, etcetera), focusing more on the treatment service (e.g. Grady & Brodersen, 2008; Levenson et al., 2014; Reimer & Mathieu, 2006).

The aim of this paper is to expand on the knowledge about how treatment contributes to ISOs’ desistance processes in the early stages, with a departure from participants’ own experiences by employing in-depth interviews. In this sense, my ambition is to further illuminate and interconnect the strands in the research field by studying the treatment process as an experiential phenomenon in combination with a contextualized, practice-oriented lens. The research question guiding the study was: how do ISOs experience participation in a sex offender treatment program and how do they experience potential change? Treatment experience was examined specifically in relation to the influence of close social relationships, societal influence, and identity.

## Perspectives on Identity Work and Change in Sex Offender Treatment

In this section, the theoretical perspectives underpinning the analysis will be presented, as well as their application to previous empirical findings related to desistance, treatment programs, and the ISO population.

### Active Responsibility

Accepting responsibility for the offense is often conceptualized as the opposite of denial and justifications (Sykes & Matza, 1957), which has been a very popular sex offender treatment target (Ware & Mann, 2012). Nonetheless, the concept of responsibility, like denial, is controversial and there is no evidence that overcoming denial would reduce re-offending (Hanson & Morton-Bourgon, 2005; Ware & Mann, 2012). Instead, confrontational therapist styles focusing on denial may hinder treatment engagement and result in dropouts, lack of treatment progress, and stigmatized (as opposed to re-integrative) shame, which do not promote successful re-entry into the community (Braithwaite, 2020; Marshall et al., 2009; Ward & Marshall, 2007; Ware & Mann, 2012). Maruna and Mann (2006) suggest the term *active responsibility* as a way forward (building on the ideas of Bovens [1998], as cited in Maruna & Mann [2006]). This concept entails a “future oriented and forward thinking, focusing on what needs to be done in order to make good or make amends or make it right” (Maruna & Mann, 2006, p. 167).

Arguably, active responsibility can promote prosocial change and desistance. However, it has been proposed this requires moving from shame to guilt (Marshall et al., 2009) (in this case, guilt is understood as an emotion, rather than guilt in a legal sense).

### (Micro-) Turning Points

Life course theories of crime stress the importance of individual-environment interaction (Sampson & Laub, 2005) and turning points is a concept commonly applied in desistance research (Carlsson, 2012). According to Carlsson, (2012, p. 3):

A turning point thus constitutes a change in the life course, which, in turn, constitutes a change in the individual’s offending. It is not employment, marriage, military service, residential change or other changes in themselves that bring about desistance, but rather the way such changes under certain circumstances can bring about other changes, which are theoretically understood as central for the desistance processes to emerge.

Hence, change is mediated by changes in routine activities, social control, self-image, or identities, as well as the previously mentioned concept of active responsibility (Carlsson, 2012). Different turning points are often interdependent on each other. In this study, I argue that ISOs’ identity work and early desistance should be studied in close relation to the context, i.e. the treatment itself, as well as the social and societal context.

In qualitative research, turning points are commonly studied retrospectively, in (confirmed) desisting offender’s accounts. In this study, the concept is temporally closer to the experienced turning points and, specifically, in relation to specific events during the treatment process. I choose to call them *micro*-turning points to denote they are concerned with smaller, yet crucial chain of events or insights (in comparison to radical life events such as role transitions). Nevertheless, it is also used more traditionally by referring to radical turning points, such as identity shifts (which may be enabled by micro-turning points). The identity work involved in such transformation is analyzed using the term narrative identity.

### Narrative Identity

Identity transformations can constitute a turning point (Maruna & Roy, 2007). The study of identity transformation in desistance processes has primarily been applied in a general offender population (e.g. Maruna, 2001). Positive subjective scripts, or identities (such as being a “family-man”) can predict decreased risk of recidivism (LeBel et al., 2008), desistance (Rocque et al., 2016), and improvements in mental health (Adler, 2012), suggesting possible causality. However, as Sampson and Laub (2016, p. 330) argue, it may not be a fact that identity transformation or changes in how people *think* changes their behavior, it could be the opposite: “*behavior* [italicized by author] changes identity”. This is consistent with the theoretical assumptions underlying the treatment program in this study: both cognitive theory and learning theory, thus the “C” and the “B” in Cognitive Behavioral Therapy (CBT), are considered important for treatment-induced change. Accordingly, treatment components should target cognitive and emotional processes as well as behavioral ones.

Identity issues in the ISO population have been studied in relation to labeling (Clarke et al., 2013) or other treatment group members (Colquhoun et al., 2018), probation supervision (Digard, 2014) or a specific treatment component such as the autobiography (Waldram, 2007) as well as redemption and (re)habilitation scripts (Kras, 2022; Mullins, 2019; Victor & Waldram, 2015). ISOs appear to be able to undergo prosocial identity transformations similar to non-sexual offenders with the assistance of treatment. However, labeling and stigma seem to contribute to somewhat mixed findings regarding the relationship between prosocial scripts and desistance (Kras, 2022). Knowledge on ISOs’ early identity transformations through treatment from an experiential perspective is scarce.

The overarching theoretical tool used in this paper, relating to the notion of identity transformation, is the concept of *narrative identity*: a person’s “internalized and evolving life story, integrating the reconstructed past and imagined future to achieve some degree of unity and purpose” (McAdams & McLean, 2013 p. 233). Narrative identity, or narrative schemas, has been employed in different research fields (e.g. Adler et al., 2017; Ibarra & Barbulescu, 2010; McAdams & McLean, 2013) and to some extent in research on ISOs (e.g. Digard, 2014; Hamilton, 2017; Kras, 2022; McAlinden et al., 2017; Mullins, 2019; Waldram, 2008). The narrative identity is constructed from the psychological, cultural, and social resources available to the individual and highlights fundamental values in one’s life (Ward & Marshall, 2007). The construction of a new prosocial (non-offending) narrative identity reflects a dynamic interactive process of identity work in which storytelling and is central.

The development of a new, non-offending narrative identity can be understood as a turning point and such a transformation also relies on active responsibility. Maruna and Mann (2006) claim that taking responsibility to overcome obstacles and solve problems in one’s life, is a feature of successful desistance, hence, arguably part of the future-oriented component in the construction of a new prosocial narrative identity.

## Method

### Recruitment of Research Subjects, Interviews, and Data Analysis

In order to capture participants’ lived experiences and early desistance processes, this study employs a qualitative research design comprising in-depth individual interviews with incarcerated participants who had recently completed the new national Swedish treatment program called SEIF (Sex offender program with individual focus). The study was approved by the Swedish Ethical Review Authority (2020-04038) as well as the Swedish Prison and Probation Service. There was a purposive sampling procedure where anyone who completed SEIF was eligible for inclusion, except individuals under the age of 18, those with severe reality-altering psychiatric conditions or those assessed as high risk of violent behaviors towards professionals.^1^ Sample size was determined using the concept of information power (Malterud et al., 2016)

Participation was voluntary and no financial compensation was offered. The power situation when conducting research in prison is asymmetrical and may create fear of consequences as well as socially desirable responses. Written informed consent was obtained and both written and verbal information was provided stating that participating (or not participating) would not in any way influence their sentence conditions. Information regarding the research project as initiated by the university, thus, being independent from the Prison and Probation Service, was emphasized. The interviewees showed no signs of feeling pressured to participate in the interviews. Prison staff helped in recruiting interviewees. Interviews were conducted from June 2021 to April 2022, with most of them taking place in the prisons and some via telephone due to the Covid-19 restrictions.

Interviews were semi-structured, experiential/phenomenological (Aspers, 2009; Braun & Clarke, 2022) as well as relationally-oriented and highly adapted to the interviewees’ own narratives (rather than asking many detailed questions). The interview procedure was inspired by *teller-focused interviews*, suitable for sensitive topics (Hydén, 2014). Although interviews included narrative prompts (Adler et al., 2017), such as requests to describe a significant event, insight, or aspect in treatment perceived to be most important, the specific term turning point was not used (Carlsson, 2012). The author has a background as a licensed healthcare counselor with basic psychotherapy training and several years of experience with ISOs, community supervision and offender treatment programs. This was perceived to have facilitated a trustful, ethically and emotionally attentive interview situation, and production of rich data.

Interviews (~ 90 minutes) were audio recorded, transcribed verbatim, inductively (data-driven) coded across the dataset, and manually analyzed in NVivo. Analysis relied on reflexive thematic analysis (Braun & Clarke, 2022) where data relevant to the research question were coded using both latent (underlying meaning) and semantic (explicit meaning) codes. All interviews were conducted and analyzed by the author, however, theoretical analysis were discussed with colleagues. Names are fictive.

### Description of Treatment Program and Sample

The interviewees participated in a new risk reducing sex offender program in Sweden called SEIF, which is based on CBT and the risk- need- and responsivity model (RNR) (Bonta & Andrews, 2017). SEIF targets medium- and high-risk ISOs and is highly individualized. Treatment duration is flexible, depends on recidivism risk, and need assessment. SEIF is delivered by the Swedish Prison and Probation Service, in group or individually, in prison, or during parole or probation, predominantly by psychologists or social workers (Lindegren, 2022). The program aims to reduce the risk of re-offending and addresses various risk factors for recidivism, primarily related to relationships, sexuality, offense-supportive cognitions, and self-regulation. Initially in treatment, there is a comprehensive risk- and need assessment, including a forensic case formulation. Furthermore, valued direction (long-term life goals) is formulated. The program ends with a risk management plan for the future, and there are booster sessions during parole. There is a strong emphasis on therapeutic alliance. Denial is treated as a responsivity issue rather than a treatment need. The Swedish Prison and Probation Service is strongly characterized by rehabilitation ideals in the spirit of so-called Scandinavian exceptionalism (Pratt, 2007).

A self-reported questionnaire were handed to the interviewees after the interview, or read out aloud in telephone interviews, in order to collect background characteristics. The sample comprised 13 incarcerated male ISOs, 26–65 years old, convicted of various sexual offenses, most commonly rape and aggravated child rape. Five interviewees had one or several non-sexual offenses under the same sentence, most frequently assault. Sentence length ranged from 1.5 years to nine years (Md=5 years). Prisons of all security levels were represented. All interviewees spoke Swedish fluently, except for one of who had lived in Sweden for about a decade. A majority of interviewees were employed before being imprisoned, had completed secondary school, and had children but presently no partner. For most of them, it was their first conviction; all of them were assessed as having medium or high risk of recidivism. No one denied their crime. A majority were closely related to the victims. There were just as many adults as there were child victims. Participants mainly attended group treatment. Only two of the interviewees reported sexual sexual attraction to children.

## Analysis

**Figure 1.**
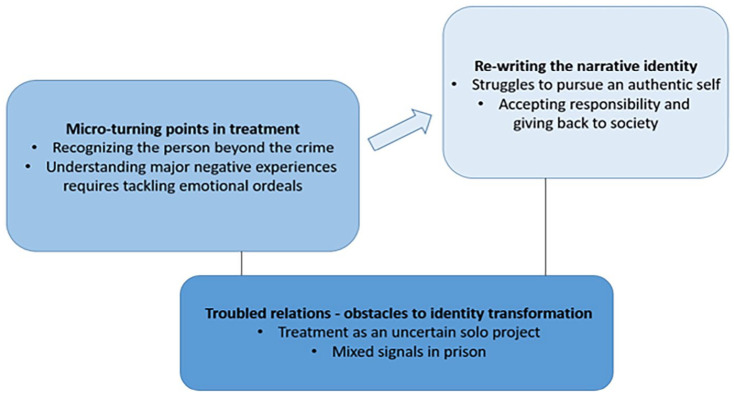
Themes and sub-themes.

The analysis demonstrates how treatment seemed to stimulate identity transformations that to some extent contributed to early desistance. The three themes illustrate various aspects of the treatment experience and perceived change as a result of treatment. Overall, interviewees felt that treatment impacted them significantly. The first theme, *troubled relations – obstacles to identity transformation* (including sub-themes) revolves around the social context of the treatment experience, and the second, *micro-turning points in treatment*, around the treatment process. The third theme, *re-writing the narrative identity*, illustrates the main outcomes from treatment, related to early development of new, desisting narrative identities, see figure 1.

### Troubled Relations - Obstacles to Identity Transformation

The first theme underlines social and relational challenges ISOs face during treatment in prison and post-release that may pose a threat to the development of a prosocial narrative identity.

#### Treatment as an Uncertain Solo Project

Although family and friends were positive towards treatment in almost all cases, they generally did not seem to discuss treatment which each other. Rather the topic was avoided. Johan, a young man with a quite extensive (non-sexual) violent criminal record, currently in a high security prison due to rape against an adult, recounts:

Johan: My older sisters, mom, dad, and those I communicate with from in here [prison], we don’t talk about it. We talk about the rest [non-sexual criminal problems]. So, both them and I, kind of skip it. I guess it’s shameful for all parties.

Treatment participation seemed to be lonely journey, without family support. Stigma and shame seemed to not only be internalized (Herek, 2007) by the ISO but also expanded to encompass his whole family, thus a form of collective, unspeakable shame. Hence, a barrier for social support as well as an obstacle to active responsibility.

The contrast, in relation to an interviewee who did receive support and involvement, is obvious. Mattias was convicted of rape against his ex-wife and had met a new girlfriend recently. He describes how he continuously discussed different treatment assignments with her, which built a strong sense of intimacy. When asked how this new relationship affected his treatment experience, he explains:

Mattias: It’s just positive, in that regard that I have had someone to discuss things with outside the treatment program, but also, as I mentioned previously, when you talk about these deep issues with someone else, you also receive their views and thoughts on it, which gives you a little more perspective. And I always want to hear her [partner’s] perspective, since she is the one with whom I want to spend the rest of my life. Thus, the better we can understand each other– needs, thoughts and emotions– the better the relationship.

Accordingly, involvement from close ones can promote the internal formulation of desired future relationships and the future self the ISO wants to be and, hence, the initial construction of a new narrative identity. Additionally, the involvement of his partner offered an opportunity for Mattias to take active responsibility – to actually do better, this time, in building trustful, non-violent, close relationships.

Another desistance obstacle (related to the imagined future self) several of the interviewees seemed to experience regarding social relations was extensive fear of post-release, contributing to the sense of an uncertain future. The constant latent threats to one’s life and health, related to the “sex offender” stigma (Tewksbury, 2012), as well as broken relationships were salient in their accounts. As Karl, a middle-aged man, convicted twice of child sexual abuse, puts it: “I haven’t been on the outside since 2017, so I don’t know how things are, really. All I know is I am serving a sentence in here and outside, I will serve a lifetime sentence.”

It seemed treatment had not helped the interviewees to resolve these issues adequately, leaving them with ongoing anxiety. One of them, Håkan, who did not want to disclose his offense, describes how he wanted to take active responsibility:

Håkan: I WANT^2^ to be there for people, so that they can have the opportunity to confront me with all their questions. I will not be able to answer everything, but at least I am going to do the best I can.

However, he was not sure how this could be achieved. SEIF, like many sex offender treatment programs, is centered on individual rehabilitation and does not include families or close ones in treatment sessions. Such a component could possibly mitigate some of these psychosocial obstacles to a prosocial identity transformation.

#### Mixed Signals in Prison

There were signs in the data of features related to non-enabling prison environments (Johnson & Haigh, 2011) thus, aspects of the prison environment that interfere with rehabilitation (cf. Levenson et al., 2023). Research on the ISO population in prison suggests that prisons communicate a stigmatizing and shaming message to ISOs, rather than a message that facilitates more constructive emotions such as guilt and remorse (Ievins, 2023). This finding was corroborated in the present study. A few interviewees claimed they were treated in degrading ways by some prison staff (not SEIF-therapists), creating mixed signals that resulted in confusion. A middle-aged, first time-convicted, interviewee describes this experience:

I have never been in a more vicious circle than the one I am in right now, in some aspects. Now, I am not talking about meeting people in here who are convicted; it is actually staff behaving really badly. [. . .] And I don’t understand, is there a purpose to this? [. . . .] Are we supposed to not feel safe anywhere? I don’t think this goes hand in hand with SEIF or any other of the activities here that are supposed to “break vicious circles” [paraphrasing the slogan of the Prison and Probation Service]. And it shouldn’t be what the management at such a place [prison] would want, arguably?

The interviewees also described a non-secure environment and that is was impossible to disclose violent incidents (from other inmates) to prison staff as this would result in becoming a snitch, leading to further violence or threats. Hence, negative or violent relationships in prison seemed to block the process of building a new stable, secure, and prosocial identity. The mix of stigmatization of ISOs in prison (e.g., from other inmates) and self-hate is demonstrated in the excerpt from James, convicted of intimate partner (sexual) violence. It also illustrates “therapeutic talk”, thus, how the participants made sense of their experiences by using theoretical notions from the SEIF program:

There is nothing you [other inmates] can say to me because I already feel resentment towards myself. So you can call me what you want, say what you want. It doesn’t change. . . because you feel self-hatred. In the program you learn there are four types of shame, it’s called “the compass of shame.” There are those who judge others; they think they are better than them because their offense is better. Regardless, what you’ve done is still bad! It hasn’t got to do with the degree [of the offense]; you have still done something bad and in the end. . . And then we have those who hide and don’t want to talk about it [the offense]. That’s that kind of shame, and then we have another one; I don’t quite remember what it was. . .? But then there is the fourth: the ones that attack themselves for what they have done. And I was there a lot. I actually wanted to go to a high security prison; I said, “send me to the worst prison you’ve got.”

Narrative identities are constructed from social and cultural resources (Ward & Marshall, 2007). Accordingly, the theme of troubled relations demonstrates how lack of social support, non-enabling prison environment, e.g., therapists and other prison staff not working in the same, rehabilitation-oriented direction, as well as (anticipated) stigma constitute barriers to the imagined future desisting self (cf. Harris et al., 2019).

### Micro-turning Points in Treatment

This theme illustrates constructive, significant events or insights: *micro-turning points*, i.e., how the interviewees perceived themselves to have changed, as well as the features of treatment that seemingly facilitated this process.

#### Recognizing the Person Beyond the Crime

A majority stated, somewhat surprised, that they were recognized as a person beyond their crime (cf. Blagden et al., 2016) and emphasized the secure and stable personal alliances between themselves and the therapists, as well as between the group-participants themselves, consistently shown to be important for treatment outcomes (Marshall, 2005; Wampold, 2015). The significance of the therapeutic alliance and relational processes, such as building trust, is emphasized in the SEIF manual. This closeness and unity (McAdams & McLean, 2013) were characterized by warmth, trust, and genuine concern, exceeding what might be expected from professionals (cf. Ugelvik, 2022). Emil, convicted of child sexual abuse, describes such a transformative process in treatment: “They didn’t just sit there like ‘psychologists’. I felt I was held by them somehow. That there was nothing, ‘you don’t need to worry about anything, just tell us what you feel’.”

This holistic, person-centered experience included perceived initial changes as well. Hamid, a young man with an extensive, primarily non-sexual, criminal record, says:

Hamid: Thoughts of revenge, aggression and violent thoughts; I had those daily. And it’s been so liberating, you know; no longer thinking that way. I don’t know how to explain this; it’s just a liberating feeling to focus on the here and now and the future, not just lying there ruminating about the past.

Accordingly, being able to reconcile the past and regulate emotions, enabled an optimistic outlook of an imagined future self, a component of a narrative identity promoting desistance (Maruna, 2001; Ward & Marshall, 2007).

#### Understanding Major Negative Experiences Requires Tackling Emotional Ordeals

This sub-theme illustrates the actual change process in treatment and what could be interpreted as micro-turning points in this process, supposedly facilitated by being seen as a person and not just a “sex offender”. Most interviewees described the re-surfacing of previous trauma or confrontation of guilt and shame in the initial treatment phase as extremely difficult. Anders, convicted of long-term sexual abuse of his children, answers the question about the most challenging part of treatment: “Well, talking about my [criminal] deeds. I mean, well, that was [gets very emotional and uncomfortable] horrible. It was horrible. It took time before I could sort of talk about them, actually”.

The micro-turning point of being able to overcome this emotional ordeal opened up for the subsequent micro-turning point, which a majority of the interviewees expressed as most important from the treatment experience. Specifically, being able to understand why they committed the crime, as well as understanding the impact of childhood (and some adulthood) experiences on later life (cf. Levenson et al., 2023). Being able to make sense of their negative self-esteem, masculinity, and one’s own neutralization techniques were also highlighted. Tobias, a young man convicted of several offenses against multiple victims, mostly children, expresses it like this:

Tobias: But I realize that I have a completely different understanding of it today, to why I ended up here. I didn’t think I could find out so much about myself and going through such a long personal development as I have done in the program. So, it has been amazing, really. To be able to understand, in depth too. And not just sort of understand that “it could have been because of this” and “I’ve felt like this previously in my life” and lived this life and then, it has led to my crimes. But also to understand more deeply like, WHY and why I lived like that, and what was it that MADE me live like that? So, yes, that’s been very good, actually. [. . .] My worst nightmare would be if I was not really able to grip WHY I committed my crimes, I think. Not understanding, maybe. Because then, I think, it would have been easy to, then the risk of doing it again increases, I believe. Or, at least that you get caught up in unhealthy life patterns.

In this regard, provided one could overcome the emotional ordeal, the autobiographical phase of treatment did not seem to result in confusion or conflicts between “experiential and forensic truths” (Waldram, 2007, p. 166) caused by specific institutional demands of disclosures (see also Maruna & Mann, 2006) demonstrated in previous research (Kruse, 2020; Waldram, 2008). Hence, the case where ISOs are being reprimanded if they display any kind of neutralizations. This finding might be due to the fact that SEIF does not target denial or include certain disclosure requirements; rather, it emphasizes collaboration and addressing risk factors for recidivism. Instead, as the excerpt indicates, this exploratory narrative processing (McAdams & McLean, 2013) contributing to a self-understanding, where the past is re-integrated into a coherent story, provided significant meaning. Hence, it was possibly a turning point for Tobias.

### Re-writing the Narrative Identity

A plausible interpretation of the data is that the micro-turning points and holistic treatment environment demonstrated in the previous theme gave rise to outcomes best understood as a transformation towards a new narrative identity.

#### Struggles to Pursue an Authentic Self

Several interviewees reported that superficial things in life were no longer important post treatment and a majority described the development of a new authentic self with close relationships as a guiding value both during and post treatment. This is consistent with the emphasis in SEIF on valued direction, i.e., long-term life goals (Hayes et al., 1999), as well as a change towards active responsibility.

Hamid: The first sessions, I had to take a break you know. It was hard. And then I had that focus – that I should think about myself, my family, and my children. So, when it was the most difficult, I had that in my mind. That was my motivator. Why I kept going.

This strive for relatedness, one of several primary goods, i.e. intrinsically beneficial experiences or activities (Ward & Marshall, 2007), seemed to guide the treatment process, also for those who experienced broken relationships. Some of them also described they developed an internal motivation during treatment, as opposed to the external motivators, such as receiving better conditions for aspects of the prison sentence, which contributed to them entering the treatment program. Thus, responsibility shifted from passive to more active forms.

Most interviewees reported a sense of being a better person post treatment, opening up to accept oneself as a person, possibly worthy of existence. Nevertheless, several interviewees struggled with maintaining a positive healthy self-image and occasionally seemed to want to convey they were not really a “sex offender” (Jacobsson & Åkerström, 2013; Presser, 2004). At the same time, many of them expressed the same negative emotions towards ISOs as the public. Accepting the stigma as part of one’s self-concept, may entail difficulties integrating the deeds into a positive and healthy self-image and can result in internal conflicts (Maruna & Mann, 2006; Ware & Mann, 2012). This constitutes obstacles to both active responsibility and a prosocial narrative identity. As Gunnar, convicted of abusing his children, describes this struggle:

Gunnar: I blame myself for having done this. That I failed so totally. I mean, it’s not ME. It’s like there is a completely different person who committed this. However, like I said, at this point, I am able to talk about it.

Gunnar’s statement suggests that silencing shame has been replaced with a more constructive emotion – guilt. Such an emotional transition is an intermediate treatment goal according to the SEIF manual, and shame is explicitly addressed in the program. Even though he has not yet reconciled these two identities, this can be interpreted as a starting point of a life-long struggle to pursue a process of change and a construction of a new, desisting narrative identity (cf. Todd-Kvam & Todd-Kvam, 2022).

#### Accepting Responsibility and Giving Back to Society

Another aspect most of the interviewees reported as crucial in the experience of treatment was how it allowed for them to accept responsibility and give back to society, emphasizing active responsibility in the future-oriented component of the developing new narrative identity. They wanted to be accountable for their actions, for their future self and life, for their family, and in some cases for the victims. Some of them discussed how to eventually apologize to their victim(s). Karl recounts:

Yeah, I have talked to the psychologists about that. Because I was planning to say “forgive me,” but they didn’t think that was a good idea at all; instead, an apology is sufficient. As they framed it; I can never force them to forgive me, because then they have to choose “no, I don’t forgive him” or “I forgive him.” I don’t want to put them in front of that choice, so to speak.

They were also very concerned about giving something back to society as a way to redeem themselves and make amends (cf. Kras, 2022; Maruna, 2001). Almost all the interviewees stated that volunteering for the research interview was one way for them to help others and give something back to the community. As Anders says: “If it helps to prevent even ONE criminal act, or that information is spread or anything like that, it is worth it. Any day of the week”. This finding also aligns with valued direction in SEIF, thus, treatment seemed to facilitate actions that were in line with guiding prosocial values, i.e., the person the ISO wants to be (e.g., someone who takes responsibility and contributes to society).

## Concluding Discussion

The main contribution of this paper is the elucidation of how treatment seemed to have contributed to the early development of a new, desisting narrative identity and what challenges this process entailed regarding social relations. The treatment experience was often described as transformative, sometimes even life changing, with them using phrases such as *it saved me* or *it’s the best thing that has happened in my life*. Supposedly, crucial micro turning points in treatment were facilitated by being recognized as a person, not just a “sex offender” (cf. Blagden et al., 2016). Such interdependent micro-turning points included overcoming emotional ordeals during the initial phase and subsequently making sense of negative life experiences and the offense. This understanding as well as being able to take active responsibility seemed to promote identity reconciliation, although this should be understood as a continuous process with recurrent negotiations, in particular in social interactions (McAdams & McLean, 2013; Ward & Marshall, 2007). These new, early desisting narrative identities, i.e. coherent redemptive internalized life-stories (cf. Kras, 2022; Maruna, 2001) are possibly supportive of desistance and something which can be understood as a turning point in itself. The findings of a transformed identity are, in several aspects, in line with previous research on ISOs employing other study designs (e.g. Harris, 2014; Kras, 2022; Mullins, 2019; Victor & Waldram, 2015).

Improved social relationships seemed to serve as fundamental motivators as well as perceived outcomes of treatment engagement, supporting the notion that relational experiences and significant meetings play a central role in the co-creation of positive self-narratives in the desistance process (cf. Todd-Kvam & Todd-Kvam, 2022).

Findings in the current study support Harris’ (2021) arguments that ISOs’ desistance processes differ in several aspects to the ones of non-sexual offenders. The analysis does not suggest knifing of the past (Sampson & Laub, 2005, 2016) as an essential aspect of ISOs’ desistance narratives. Rather, the past appeared to be an important object of investigation and sense making. Being able to comprehend, reconstruct, and re-integrate the past into a new narrative identity offered a starting point for desistance (Maruna & Roy, 2007). Similarly, the ISOs’ experiences did not relate to the need to end antisocial networks; rather, the difficulties for them seemed to comprise broken prosocial relationships they often wanted to repair. In this regard, stigma and collective shame affecting everyone around the ISO seemed to block the attainment of primary human goods, in this case relatedness (Harris et al., 2019), which is essential when building a new prosocial narrative identity (Ward and Marshall 2007). Thus, in line with previous research, stigma and shame constitute clear barriers to desistance processes (Cullen et al., 2020; K. McCartan & Kemshall, 2021; Sandbukt, 2023). More research examining the role of close social relations during the later stages of ISOs’ desistance process is needed, as are investigations into stigma management for ISOs.

### Implications for Policy and Practice

The findings in the current study suggest a person-centered, holistic treatment environment can support desistance processes and prosocial identity transformation among ISOs, despite the criticism of the risk-focus evident in RNR-based treatment programs (Harris, 2021; Ward et al., 2007). In this regard, the current study did not highlight risk discourses as salient, in contrast to the study by Mullins (2019). Moreover, it supports the idea that therapeutic processes mitigate negative labeling (Willis, 2018) and promote active responsibility. This corroborates the criticism against unnecessary or even counterproductive efforts to overcome denial in treatment (Maruna & Mann, 2006; Ware & Mann, 2012), since the latter is not an objective in the treatment program studied.

A possible interpretation is that the process of improved understanding and re-integration of the past, including the offense, into a new redemptive identity was enabled by the assistance of therapists and the use of comprehensive forensic case formulations. Plausible explanations for problematic behaviors, and their solution, are important in the creation of expectations about the effectiveness of an intervention as well as goal-agreement, an essential pathway to change (Wampold, 2015). It is conceivable that professional guidance in this phase is particularly important for ISOs since driving forces behind sexual offenses are perhaps less self-explanatory than other offenses. In addition, sexual offenses entail exceptionally accentuated challenges to one’s self-concept.

Non-enabling environments in the correctional system (cf. Levenson et al., 2023; Ievins, 2023) may hinder desistance processes, as well as insufficient attention to stigma management and collective shame, which may limit opportunities for active responsibility. Since ISOs seldom have peer- or support groups similar to substance use or mental health service users, there is a need to develop additional support addressing post-release social life issues, possibly quite early in the sentencing process, which could also enhance responsivity during the treatment process.

### Methodological Considerations

Findings in the current study may be transferable (Smith, 2018) to other similar interventions or other groups subjected to significant stigmatization. However, sex offender treatment programs differ substantially even within the group of programs based on CBT and RNR (Polaschek, 2011); accordingly, the findings may not necessarily apply to other settings. Additionally, the Swedish context for ISOs may differ a lot from those in other countries, due to, for instance, a strong correctional rehabilitation culture as well as the absence of public registries and notification laws. This may affect treatment experience as well as stigma management. Furthermore, it is conceivable that the interviewees volunteering here comprised a non-representative group of individuals who were the most satisfied with the treatment; the ones offering the “correct” narrative, from an institutional perspective (cf. Waldram, 2007). This can result in a positive selection bias.

The use of a self-reported questionnaire regarding background characteristics may risk socially desirable responses. Nonetheless, this procedure was deemed to increase the number of volunteers since collecting comprehensive register data can deter some ISOs from participating. The fact that most interviewees (84.6%) were convicted of offenses against a closely related adult or child may contribute to the frequent occurrence of identity- as well as relationship-related themes. Hence, abusing, and thus seriously hurting, your own child or partner might create exceptionally strong inner conflicts as well as accentuated social challenges and broken relationships in the closest circle of family and friends.

Life stories or accounts of individuals do not represent the “truth” (Adler et al., 2017) because of the bias that humans, in general, are prone to in understanding causes of their own actions (Sampson & Laub, 2016). Rather, they describe how individuals make sense of their experiences. Interviewees sometimes used therapeutic talk; thus, their accounts were highly influenced by the therapeutic ideas underpinning the treatment program. Hence, experiences are highly influenced by discourses.

Even though the interpretation indicates an early development of narrative identities associated with desistance, only the future can tell whether the interviewees will actually desist or not. Since all interviewees were incarcerated, they had not yet encountered severe high-risk situations, which typically occur outside of prison.

## Notes

1. Two interviewees had a few sessions of SEIF left; one had attended the precursor of SEIF, called ROS (Relations- and Co-existence program, author’s translation), and another attended a combination of SEIF and ROS.

2. Words expressed with emphasis by interviewees are spelled with capital letters.
